# Primary immune regulatory disorders (PIRD): expanding the mutation spectrum in Turkey and identification of sixteen novel variants

**DOI:** 10.1007/s12026-024-09477-6

**Published:** 2024-04-22

**Authors:** Ayca Aykut, Asude Durmaz, Neslihan Karaca, Nesrin Gulez, Ferah Genel, Fatih Celmeli, M. Tuba Cogurlu, Mediha Akcan, Dilek Cicek, Funda Erol Cipe, Ayca Kiykim, Alisan Yıldıran, Kursad Unluhizarci, Sara Sebnem Kilic, Guzide Aksu, Omur Ardeniz, Necil Kutukculer

**Affiliations:** 1 Department of Medical Genetics, Faculty of Medicine, Ege University, Bornova, Izmir, Turkey; 2 Department of Pediatric Health and Diseases, Department of Pediatric Immunology, Faculty of Medicine, Ege University, Izmir, Turkey; 3 Pediatric Immunology and Allergy Diseases, Saglık Bilimleri University, Uz Pediatric Diseases and Surgery Training and Research Hospital, Dr. Behcet, Izmir, Turkey; 4 Immunology and Allergy Diseases, Saglık Bilimleri University, Antalya Training and Research Hospital Pediatric, Antalya, Turkey; 5 Department of Pediatric Health and Diseases, Department of Pediatric Immunology, Saglık Bilimleri University, Kocaeli Derince Training and Research Hospital, Kocaeli, Turkey; 6 Department of Pediatrics, Division of Pediatric Hematology and Oncology, Faculty of Medicine, Adnan Menderes University, Aydın, Turkey; 7 Department of Pediatric Endocrinology, Faculty of Medicine, Erciyes University, Kayseri, Turkey; 8 Pediatric Immunology and Allergy Diseases, Saglık Bilimleri University Kanuni Sultan Suleyman Training and Research Hospital, Istanbul, Turkey; 9 Department of Pediatric Health and Diseases, Cerrahpasa Faculty of Medicine, Pediatric Allergy Immunology, Istanbul, Turkey; 10 Department of Pediatric Health and Diseases, Department of Pediatric Immunology, Faculty of Medicine, Ondokuz Mayıs University, Samsun, Turkey; 11 Department of Endocrinology, Faculty of Medicine, Erciyes University, Kayseri, Turkey; 12 Department of Pediatric Immunology and Rheumatology, Faculty of Medicine, Bursa Uludag University, Bursa, Turkey; 13 Department of Immunology, Faculty of Medicine, Ege University, Izmir, Turkey

**Keywords:** Next-generation sequencing, PIRD, Novel mutation

## Abstract

Human Inborn Errors of Immunity (IEIs) encompass a clinically and genetically heterogeneous group of disorders, ranging from mild cases to severe, life-threatening types. Among these, Primary Immune Regulatory Disorders (PIRDs) constitute a subset of IEIs characterized by diverse clinical phenotypes, prominently featuring severe atopy, autoimmunity, lymphoproliferation, hyperinflammation, autoinflammation, and susceptibility to malignancies. According to the latest report from the International Union of Immunological Societies (IUIS), PIRDs arise from mutations in various genes including *LYST*, *RAB27A*, *AP3B1*, *AP3D1*, *PRF1*, *UNC13D*, *STX11*, *STXBP2*, *FAAP24*, *SLC7A7*, *RASGRP1*, *CD70*, *CTPS1*, *RLTPR*, *ITK*, *MAGT1*, *PRKCD*, *TNFRSF9*, *SH2DIA*, *XIAP*, *CD27 (TNFRSF7)*, *FAS (TNFRSF6)*, *FASLG (TNFSF6)*, *CASP10*, *CASP8*, *FADD*, *LRBA*, *STAT3*, *AIRE*, *ITCH*, *ZAP70*, *TPP2*, *JAK1*, *PEPD*, *FOXP3*, *IL2RA*, *CTLA4*, *BACH2*, *IL2RB*, *DEF6*, *FERMT1*, *IL10*, *IL10RA*, *IL10RB*, *NFAT5*, *TGFB1*, and *RIPK1* genes. We designed a targeted next-generation sequencing (TNGS) workflow using the Ion AmpliSeq™ Primary Immune Deficiency Research Panel to sequence 264 genes associated with IEIs on the Ion S5™ Sequencer. In this study, we report the identification of 38 disease-causing variants, including 16 novel ones, detected in 40 patients across 15 distinct PIRD genes. The application of next-generation sequencing enabled rapid and precise diagnosis of patients with PIRDs.

## Introduction

Inborn Errors of Immunity (IEIs) are a group of genetically and phenotypically heterogeneous inherited disorders. The identification of monogenic defects underlying IEIs has steadily increased over time [1]. In 2022, the count of known IEIs rose to 485, and the classification was updated to encompass more than 450 distinct gene defects [2]. Human IEIs are categorized into 10 groups based on shared pathogenesis and immune system components involved [2, 3]. As per the European Society for Immunodeficiencies (ESID), Primary Immune Regulatory Disorders (PIRDs) account for around 5.3% of IEIs, with 45 disease-causing genes currently categorized across four classes as follows: hemophagocytic lymphohistiocytosis (HLH), susceptibility to EBV, syndromes involving autoimmunity, and immune dysregulation with colitis [2–4] (refer to Table 1).

**Table 1:** Diseases of immune dysregulation. A Hemophagocytic lymphohistiocytosis and EBV susceptibility. B Syndromes with autoimmunity and others

A. Hemophagocytic lymphohistiocytosis and EBV susceptibility			
HLH		Susceptibility to EBV	
Hypopigmentation	Familial hemophagocytic lymphohistiocytosis syndromes	RASGRP1 deficiency *RASGRP1 A*R	EBV-associated HLH
Chediak Higashi syd. *LYST* AR	Perforin deficiency (FHL2) *PRF1* AR	CD70 deficiency *CD70 A*R	XLP1 *SH2DIA* XL
Griscelli syd type 2 *RAB27a* AR	UNC13D/Munc13-4 deficiency (FHL3)*UNC13D* AR	CTPS1 deficiency *CTPS1 A*R	XLP2 *XIAP* XL
Hermansky Pudlak syd type 2 *AP3B1* AR	Syntaxin 11 deficiency (FHL4) *STX11* AR	RLTPR (CARMIL2) deficiency RLTPR *A*R	CD27 deficiency *CD27(TNFRSF7)* AR
Hermansky-Pudlak syd type 10 *AP3D1* AR	STXBP2/Munc18-2 deficiency (FHL5) *STXBP2* AR	ITK deficiency *ITK A*R	
	FAAP24 deficiency *FAAP24AR/AD*	MAGT1 deficiency (XMEN) *MAGT1* XL	
	SLC7A7 deficiency*SLC7A7 AR*	TET2 deficiency TET2 AR LOF	
	CDC42 deficiency (NOCARH syndrome) CDC42 AD	PRKCD deficiency *PRKCD* AR	
	RHOG deficiency AR	CD137 deficiency*TNFRSF9* AR	
B. Syndromes with autoimmunity and others			
Syndromes with autoimmunity			Immune dysregulation with colitis (IBD)
Increased CD4-CD8-TCR α/β double negative (DN) T cells?			IL-10 deficiency *IL10* AR
Yes	No: regulatory T-cell defect?		IL-10Ra deficiency *IL10RA* AR IL-10Rb deficiency *IL10RB* AR
	No	Yes	
ALPS	APECED (APS-1) *AIRE* AR/AD	IPEX *FOXP3* XL	IL21 deficiency *IL21*AR
ALPS-FAS. *TNFRSF6* AD	ITCH deficiency *ITCH* AR	CD25 deficiency *IL2RA* AR	NFAT5 haploinsufficiency *NFAT5* AD
ALPS-FASLG *TNFSF6* AR	ZAP-70 *ZAP70* AR (LOF/GOF)	CTLA4 deficiency *CTLA4 A*D	TGFB1 deficiency *TGFB1* AR
ALPS-Caspase10 *CASP10* AD	Tripeptidyl Peptidase II deficiency *TPP2* AR	BACH2 deficiency*BACH2* AD	RIPK1 deficiency *RIPK1* AR
	JAK1 GOF *JAK1* AD	*STAT3* GOF mutations*STAT3 AR*	ELF4 deficiency *ELF4 XL*
	Prolidase deficiency *PEPD* AR	CD122 deficiency *IL2RB AR*	
	Caspase 8 *CASP8* AR	FERMT1 deficiency *FERMT1 AR*	
	FADD deficiency *FADD* AR	DEF6 deficiency *DEF6 AR*	
	SOCS1 deficiency *SOCS1* AD	LRBA deficiency *LRBA* AR	
		IKAROS *GOF IKZF1* AD	

*ALPS* autoimmune lymphoproliferative syndrome, *HLH* hemophagocytic lymphohistiocytosis, *AD* autosomal dominant transmission, *XL* X-linked transmission, *AR* autosomal recessive transmission, *EBV* Epstein–Barr virus, *AD* autosomal dominant transmission, *XL* X-linked transmission, *AR* autosomal recessive transmission, *GOF* gain of function, *LOF* loss of function

PIRDs constitute a growing collection of diseases arising from gene defects across various immune pathways, notably those affecting regulatory T-cell function. Unlike classical IEIs, PIRDs may present with autoimmune symptoms like cytopenia, enteropathy, and dermatitis as initial indications of the disorder [4–6].

Though IEIs are rare globally, they exhibit higher prevalence in regions with significant consanguinity due to the prevalence of autosomal recessive IEIs [7]. The exact incidence of IEIs in Turkey remains uncertain due to the absence of comprehensive national disease registries and limited incidence studies. Nonetheless, given the elevated rates of consanguineous marriages in our country, the incidence of both IEIs and PIRDs is anticipated to surpass the reported Figs. [8–10].

The advent of Next-Generation Sequencing (NGS) has ushered in a new era in genomics research, offering unparalleled capabilities in terms of speed, cost-effectiveness, and throughput. In the context of PIRD, understanding molecular genetics is essential, and NGS stands as a powerful tool to unravel the complexities associated with these disorders. NGS significantly enhances diagnostic accuracy by providing a comprehensive genetic profile, minimizing the risk of misdiagnosis. This precision in diagnosis is paramount for effective patient management and timely interventions.

The substantial surge in recognized monogenic inborn errors of immunity disorders in recent years can be attributed to the wider adoption of next-generation sequencing (NGS) technology [11–14]. The application of multi-gene panels for targeted sequencing, especially for specific disease groups like PIRDs, has facilitated swift diagnoses through next-generation sequencing methods [14].

This study aims to examine the spectrum of PIRD gene mutations in Turkish patients, thereby extending our understanding of molecular genetics in this context.

## Material method

Patients (*n* = 40) diagnosed with Primary Immune Regulatory Disorders (PIRDs) at the Medical Genetics Department between 2018 and 2021 were enrolled in this study. Comprehensive clinical and laboratory findings are summarized in Table 2. The study received approval from the ethics committee, and no external financial support was obtained. Written informed consent was secured from the parents of the participants.

**Table 2:** Clinical classification features and disease-causing variants identified in PIRD patients

Patient	Consanguinity	Gender	Age	Gene	Transcript	PIRD classification	Clinical presentation	Inheritance	Zygosity	cDNA	Protein	Novel/previously described	ACMG
1	Yes	F	1	*LYST*	NM_000081.3	HLH	Recurrent cutaneous and systemic pyogenic infections	AR	Homozygous	c.6077_6078insA	p.Tyr2026Ter	Previously described	Likely pathogenic
2	Yes	F	11	*LYST*	NM_000081.3	HLH	Septic sacroiliitis	AR	Homozygous	c.52C > T	p.Arg18Trp	Previously described	VUS (PM2,PM5)
3	Yes	M	1	*LYST*	NM_000081.3	HLH	Recurrent infections	AR	Homozygous	c.10699C > T	p.Gln3567Ter	Novel	Pathogenic
4	Yes	F	1	*LYST*	NM_000081.3	HLH	Hair hypopigmentation	AR	Homozygous	c.10423 T > C	p.Ser3475Pro	Novel	VUS (PM2)
5	Yes	F	1	*RAB27A*	NM_183235.3	HLH	Hair hypopigmentation	AR	Homozygous	c.53_54delCT	p.Ser18fs	Previously described	Pathogenic
6	Yes	M	20	*STXBP2*	NM_006949.4	HLH	EBV infection	AR	Homozygous	c.1280-1G > C	-	Previously described	Pathogenic
7	No	M	12	*MAGT1*	NM_001367916.1	Susceptibility to EBV	Hodgkin lymphoma	XL	hemizygous	c.244C > T	p.Gln82Ter	Novel	Likely pathogenic
8	Yes	M	23	*SH2D1A*	NM_002351.4	Susceptibility to EBV	ALPS?	XL	Hemizygous	c.164G > A	p.Arg55Gln	Previously described	Likely pathogenic
9	No	M	28	*SH2D1A*	NM_002351.4	Susceptibility to EBV	Hyper IgM	XL	Hemizygous	c.163C > T	p.Arg55Ter	Previously described	Likely pathogenic
10	No	M	2	*XIAP*	NM_001167.3	Susceptibility to EBV	Enteropathy, splenomegaly	XL	Hemizygous	c.482A > G	p.Tyr161Cys	Previously described	VUS (PM2,NBP4)
11	No	F	8	*CASP10*	NM_032977.3	Syndromes with autoimmunity	ALPS?	AD	Heterozygous	c.1297G > A	p.Glu433Lys	Previously described	VUS (PM2)
12	No	M	4	*FAS*	NM_032977.3	Syndromes with autoimmunity	ALPS?	AD	Homozygous	c.869C > T	p.Ala290Val	Novel	VUS (PM2,PP2)
13	Yes	M	5	*FAS*	NM_032977.3	Syndromes with autoimmunity	N/A	AD	Heterozygous	c.457A > T	p.Ile153Phe	Novel	VUS (PM2,PP2)
14	No	M	2	*FAS*	NM_000043.6	Syndromes with autoimmunity	Neutropenia	AD	Heterozygous	c.452A > G	p.His151Arg	Novel	VUS (PM2,PP2)
15	Yes	M	10	*LRBA*	NM_001364905.1	Syndromes with autoimmunity	ALPS?	AR	Homozygous	c.2496C > A	p.Cys832Ter	Novel	Likely pathogenic (PVS1,PM2)
16	Yes	F	49	*LRBA*	NM_001364905.1	Syndromes with autoimmunity	Splenomegaly	AR	Homozygous	c.2165G > A	p.Arg722His	Previously described	VUS (PM2,PP3)
17	Yes	M	15	*LRBA*	NM_001364905.1	Syndromes with autoimmunity	Autoimmune hemolytic anemia, splenomegaly	AR	Homozygous	c.5505delT	p.Ile1836fs	Previously described	Likely pathogenic
18	Yes	M	15	*LRBA*	NM_001364905.1	Syndromes with autoimmunity	Hypogammaglobulinemia	AR	Homozygous	c.5123 T > G	p.Leu1708Arg	Novel	VUS (PM2)
19	Yes	M	32	*LRBA*	NM_001364905.1	Syndromes with autoimmunity	Hypogammaglobulinemia	AR	Homozygous	c.6834delA	p.Glu2278fs	Novel	Likely pathogenic (PVS1,PM2)
20	Yes	F	20	*LRBA*	NM_001364905.1	Syndromes with autoimmunity	Hepatosplenomegaly autoimmune cytopenia	AR	Homozygous	c.7943C > A	p.Ser2648Ter	Previously described	Likely pathogenic
21	No	M	38	*CTLA4*	NM_005214.5	Syndromes with autoimmunity	Autoimmune hemolytic anemia	AD	Heterozygous	c.436G > A	p.Gly146Arg	Previously described	VUS (PM2,PP5)
22	No	M	23	*CTLA4*	NM_005214.5	Syndromes with autoimmunity	ALPS	AD	Heterozygous	c.19C > T	p.Gln7Ter	Novel	Likely pathogenic (PVS1,PM2)
23	No	M	25	*CTLA4*	NM_005214.5	Syndromes with autoimmunity	Autoimmune polyglandular syndrome	AD	Heterozygous	c.495_496delinsAT	p.Trp165Ter	Novel	Likely pathogenic (PVS1,PM2)
24	No	M	14	*CTLA4*	NM_005214.5	Syndromes with autoimmunity	ALPS	AD	Heterozygous	c.60G > A	p.Trp20Ter	Previously described	Pathogenic
25	No	M	19	*CTLA4*	NM_005214.5	Syndromes with autoimmunity	Hepatosplenomegaly, enteropathy	AD	Heterozygous	c.518G > A	p.Gly173Glu	Previously described	VUS(PM2)
26	No	M	30	*STAT3*	NM_139276.2	Syndromes with autoimmunity	Autoimmune hemolytic anemia	AD	Heterozygous	c.973C > T	p.Arg325Trp	Previously described	VUS (PM2,PM1,PP2,PP3)
27	No	F	7	*STAT3*	NM_139276.2	Syndromes with autoimmunity	Autoimmune hemolytic anemia, hepatosplenomegaly	AD	Heterozygous	c.1981G > T	p.Asp661Tyr	Previously described	Likely pathogenic
28	No	F	3	*STAT3*	NM_139276.2	Syndromes with autoimmunity	Hypereosinophilia	AD	Heterozygous	c.1295 T > C	p.Val432Ala	Novel	VUS (PM2,PM1,PP2,PP3)
29	No	F	12	*STAT3*	NM_139276.2	Syndromes with autoimmunity	Hypereosinophilia	AD	Heterozygous	c.1144C > T	p.Arg382Trp	Previously described	Pathogenic
30	No	M	15	*STAT3*	NM_139276.2	Syndromes with autoimmunity	ALPS	AD	Heterozygous	c.31G > A	p.Asp11Asn	Novel	VUS (PM2,PP1)
31	No	M	45	*STAT3*	NM_139276.2	Syndromes with autoimmunity	Hepatosplenomegaly	AD	Heterozygous	c.1324G > C	p.Glu442Gln	Novel	VUS (PM2,PM1,PP2)
32	No	M	18	*STAT3*	NM_139276.2	Syndromes with autoimmunity	Agranulocytosis	AD	Heterozygous	c.454C > T	p.Arg152Trp	Previously described	Likely pathogenic
33	Yes	M	3	*AIRE*	NM_000383.4	Syndromes with autoimmunity	Chronic mucocutaneous candidiasis	AR	Homozygous	c.769C > T	p.Arg257Ter	Previously described	Pathogenic
34	Yes	M	30	*AIRE*	NM_000383.4	Syndromes with autoimmunity	Chronic mucocutaneous candidiasis	AR	Homozygous	c.769C > T	p.Arg257Ter	Previously described	Pathogenic
35	Yes	F	14	*AIRE*	NM_000383.4	Syndromes with autoimmunity	Chronic mucocutaneous candidiasis	AR	Homozygous	c.769C > T	p.Arg257Ter	Previously described	Pathogenic
36	Yes	M	2	*AIRE*	NM_000383.4	Syndromes with autoimmunity	Chronic mucocutaneous candidiasis	AR	Homozygous	Exon 2–3-4 deletion		Novel	Likely pathogenic
37	Yes	M	3	*ZAP70*	NM_001079.3	Syndromes with autoimmunity	Recurrent CMV infection	AR	Homozygous	c.1448C > T	p.Ser483Phe	Novel	Likely pathogenic (PM2,PP3)
38	No	M	6	*FOXP3*	NM_014009.3	Syndromes with autoimmunity	Enteropathy	XL	Hemizygous	c.506G > A	p.Cys169Tyr	Previously described	VUS(PM2)
39	No	M	3	*FOXP3*	NM_014009.3	Syndromes with autoimmunity	N/A	XL	Hemizygous	c.1150G > A	p.Ala384Thr	Previously described	Pathogenic
40	No	F	6	*IL10RB*	NM_000628.5	immune dysregulation with colitis (IBD)	Chronic diarrhea	AR	Homozygous	c.477G > A	p.Trp159Ter	Previously described	Pathogenic

*M* male, *F* female, *PIRD* primary immune regulatory disorders, *AD* autosomal dominant, *AR* autosomal recessive, *XL* X-linked, *HLH* hemophagocytic lymphohistiocytosis, *EBV* Epstein–Barr virus *ALPS* autoimmune lymphoproliferative syndrome, *N/A* non available, *ACMG* American College of Medical Genetics, *VUS* variant of unknown significance

PVS1: Very strong evidence of pathogenicity Null variant (nonsense, frameshift, canonical ± 1 or 2 splice sites, initiation codon, single or multi-exon deletion) in a gene where loss of function (LOF) is a known mechanism of disease

PM2: Moderate evidence of pathogenicity, absent from controls (or at extremely low frequency if recessive) in exome sequencing project, 1000 genomes or ExAC

PM1: Moderate evidence of pathogenicity, located in a mutational hot spot and/or critical and well-established functional domain (e.g., active site of an enzyme) without benign variation

PP2: Missense variant in a gene that has a low rate of benign missense variation and where missense variants are a common mechanism of disease

PP3: Supporting evidence of pathogenicity, multiple lines of computational evidence support a deleterious effect on the gene or gene product (conservation, evolutionary, splicing impact, etc.)

DNA was extracted from whole blood utilizing the QIAamp DNA Blood Mini Kit (Qiagen, Hilden, Germany). The amount of extracted DNA was quantified employing the Qubit™ dsDNA HS assay kit on the Qubit 2.0 fluorometer (Thermo Fisher Scientific), following the manufacturer’s guidelines. Library preparation was performed using the Ion Chef System (Thermo Fisher Scientific, San Francisco, CA, USA) as per the manufacturer’s protocols. Barcoded libraries were generated from 10 ng of DNA per sample through the Ion AmpliSeq Chef Solutions (Thermo Fisher Scientific) and the Ion AmpliSeq™ Primary Immune Deficiency Research Panel v2 (Thermo Fisher Scientific). This panel comprises 5241 amplicons within 264 genes. The resulting libraries were clonally amplified onto Ion Sphere Particles (ISP) using emulsion PCR within an Ion Chef System (Thermo Fisher Scientific), following the manufacturer’s guidelines. Enriched ISPs were loaded onto 530 chips accommodating 16 samples per sequencing run. Sequencing was executed using an Ion S5 Sequencer alongside an Ion 530 Chip and an Ion 530 kit–Chef Kit (all from Thermo Fisher Scientific). The sequences were aligned to the reference genome hg19, and base calling was carried out using the Torrent Suite software. The annotated variant-calling file (VCF) was subjected to filtration in Ion ReporterTM Software, displaying only the variants relevant to the 27 PIRD-related genes (*LYST*, *RAB27A*, *PRF1*, *STX11*, *STXBP2*, *SLC7A7*, *CTPS1*, *ITK*, *MAGT1*, *PRKCD*, *SH2DIA*, *XIAP*, *FAS (TNFRSF6)*, *FASLG (TNFSF6)*, *CASP10*, *CASP8*, *FADD*, *LRBA*, *STAT3*, *AIRE*, *ITCH*, *ZAP70*, *FOXP3*, *CTLA4*, *IL10*, *IL10RA*, *IL10RB*).

The clinical significance of the novel variants was assessed using the American College of Medical Genetics and Genomics (ACMG) and Association for Molecular Pathology (AMP) recommended standards and guidelines [15]. Minor Allele Frequencies were examined through databases including NCBI dbSNP build141, 1000 Genomes Project, Exome Aggregation Consortium (ExAC), and Genome Aggregation Database (gnomAD). Information specific to disease-related variants was obtained from ClinVar and OMIM. The impact of novel variants on protein structure was evaluated using prediction tools such as GERPP, Polyphen-2, and SIFT [16–18]. Variant pathogenicity was assessed in line with ACMG recommendations. All novel genetic variants were scrutinized for their pathogenicity, inheritance mode, and clinical manifestations. Missense variants affected conserved amino acids within functional protein domains. Notably, all variants were absent in gnomAD. Finally, candidate pathogenic variants identified through NGS were validated using Sanger sequencing on an ABI PRISM 3500 DNA analyzer (Applied Biosystems), followed by segregation analysis.

## Results

The cohort consisted of 40 patients—14 pediatric and 26 adult individuals, with 28 males (70.0%) and 12 females (30%), resulting in a male-to-female ratio of 2.3 (28/12).

Allelic segregations for the identified gene variants were verified within the families. In total, 38 disease-causing variants were pinpointed across 15 distinct PIRD genes: *STAT3* (*n* = 7), *LRBA* (*n* = 6), *CTLA4* (*n* = 5), *LYST* (*n* = 4), *AIRE* (*n* = 4), *FAS* (*n* = 3), *SH2D1A* (*n* = 2), *FOXP3* (*n* = 2), *XIAP* (*n* = 1), *ZAP70* (*n* = 1), *CASP10* (*n* = 1), *RAB27A* (*n* = 1), *STXBP2* (*n* = 1), *MAGT1* (*n* = 1), and *IL10RB* (*n* = 1). Our cohort encompassed HLH (*n* = 6), susceptibility to EBV (*n* = 4), syndromes featuring autoimmunity (*n* = 29), and one patient with immune dysregulation and colitis (IBD).

In this study, we unveil 16 novel variants, including three homozygous variants (c.2496C > A (p.Cys832Ter), c.5123 T > G (p.Leu1708Arg), c.6834delA (p.Glu2278fs)) in LRBA; two homozygous variants (c.10699C > T (p.Gln3567Ter), c.10423 T > C (p.Ser3475Pro)) in LYST; one homozygous deletion in AIRE spanning exons 2 to 3; one homozygous variant (c.1448C > T (p.Ser483Phe)) in ZAP70; one homozygous variant (c.869C > T (p.Ala290Val)) and two heterozygous variants (c.457A > T (p.Ile153Phe), c.452A > G (p.His151Arg)) in FAS; three heterozygous variants (c.1295 T > C (p.Val432Ala), c.31G > A (p.Asp11Asn), c.1324G > C (p.Glu442Gln)) in STAT3; two heterozygous variants (c.19C > T (p.Gln7Ter), c.495_496delinsAT (p.Trp165Ter) in CTLA4; and one hemizygous variant (c.244C > T (p.Gln82Ter)) in MAGT1. These novel variants expand the spectrum of clinical manifestations attributed to novel PIRD variants and broaden the genetic landscape of Human Inborn Errors of Immunity.

### Genotype–phenotype correlations of the patients with novel variations

**Fig. 1:**
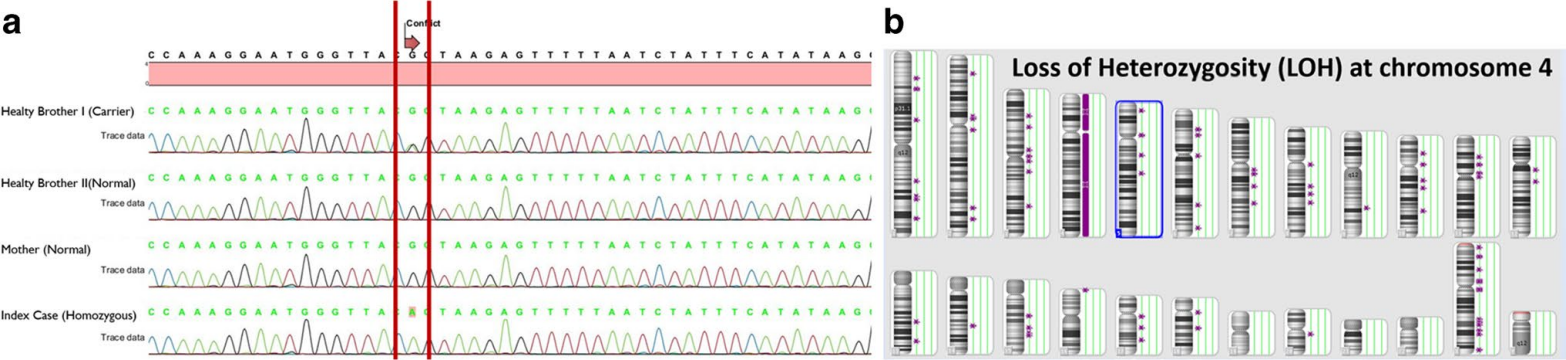
**a** Segregation analysis of the family and **b** SNP array analysis of the index

We present a 49-year-old female (case 16) diagnosed with common variable immune deficiency, recurrent respiratory tract infections, cough attacks, periodic hematuria, dysuria, and hypogammaglobulinemia (IgG < 34 mg/dl; IgA < 27 mg/dl; IgM < 17 mg/dl, IgE < 17.9 IU/ml). Over 11 years, she had asthma, while rheumatoid arthritis and hypothyroidism were diagnosed 3 years ago. Sequence analysis revealed a homozygous c.2165G > A (p.Arg722His) variation. Segregation testing was performed on the case’s mother and two healthy brothers, with one brother found to be heterozygous for c.2165G > A (p.Arg722His), while the mother and the other brother were non-carriers. The father’s DNA could not be sequenced due to his passing. Though we hypothetically considered the father as a carrier due to the autosomal recessive nature of LRBA deficiency, the circumstances remained unclear. This is inconsistent with the expected homozygous occurrence in our patient. To verify this, we confirmed genotypes via Sanger sequencing (Fig. 1a). Our findings suggested either a deletion encompassing the LRBA locus on the maternal chromosome 4 or the inheritance of two copies of the mutant maternal LRBA allele. Further analysis with an SNP array revealed loss of heterozygosity of the entire chromosome 4 (Fig. 1b).

Two male siblings with a novel LRBA variation presented with different initial symptoms: the younger one had chronic early-onset diarrhea, while the elder one had autoimmune hemolytic anemia. Both developed hypogammaglobulinemia, enteropathy, and lung involvement during long-term follow-up for the IPEX phenotype. Partial responses to immunosuppressive therapies were observed. Molecular diagnosis revealed a homozygous LRBA gene variation c.2496C > A (p.Cys832Ter), resulting in a premature stop codon. Subsequent treatment with abatacept, a target-specific molecule, showed promising results.

A novel hemizygous MAGT1 c.244C > T (p.Gln82Ter) variant was identified in a 13-year-old male patient presenting with swelling on the right side of the neck. Despite antibiotic treatment, neck lymphadenopathy persisted, leading to a referral for malignancy evaluation. B symptoms were absent during presentation, and physical examination revealed a 3 cm mobile rubbery lymphadenopathy in the right submandibular region. The patient’s parents were not consanguineous, and no family history of cancer was reported. Laboratory tests indicated normal complete blood count and biochemistry, with an LDH of 251 IU/l and an erythrocyte sedimentation rate of 9 mm/h. Excisional lymph node biopsy diagnosed the patient with classic Hodgkin’s lymphoma, nodular sclerosis type, and staging placed him in risk group TG1 under the GPOH-HD-2002 protocol. Following two cycles of OEPA treatment, a complete response was achieved, and treatment was concluded due to stage 1A and complete response attainment. Immunoglobulin levels at lymphoma diagnosis were IgA, 0.23 g/l; IgG, 6.74 g/l; and IgM, 0.23 g/l. No recurrent infections were documented, but intravenous immunoglobulin was administered during chemotherapy. Following chemotherapy, immunoglobulin levels remained low, prompting evaluation for potential immune deficiencies.

In our cohort, two novel heterozygous CTLA4 variants (c.19C > T (p.Gln7Ter) and c.495_496delinsAT (p.Trp165Ter)) were found in case 22 with cytopenia and case 23 with rheumatoid arthritis, diabetes mellitus, CVID, and alopecia totalis. These cases were treated with abatacept, yielding successful outcomes.

The preliminary diagnoses for cases with newly identified LYST variations c.10699C > T (p.Gln3567Ter) and c.10423 T > C (p.Ser3475Pro) were Chediak-Higashi syndrome.

Our cohort’s patients with novel STAT3 variants presented with c.1295 T > C (p.Val432Ala)-related hypereosinophilia, c.31G > A (p.Asp11Asn)- and c.1324G > C (p.Glu442Gln)-associated ALPS, and hepatosplenomegaly.

## Discussion

Inborn Errors of Immunity (IEIs) encompass a group of genetically and phenotypically heterogeneous inherited disorders that impede the development and/or function of the human immune system [1]. Primary Immune Regulatory Disorders (PIRDs) are linked to autoimmunity, autoinflammation, and/or disruptions in lymphocyte homeostasis [4]. Commonly, defects in T cells and their tolerance induction, B cells, immunoglobulins, and class-switch recombination, as well as genes affecting multiple cellular subsets, constitute the prevalent issues predisposing IEI patients to autoimmunity [19]. The advent of NGS technologies has revealed an expanding list of monogenic defects underlying IEIs, with diagnostic yields ranging from 15 to 79% [13].

In these 40 index patients, a total of 38 disease-causing variants (16 of which were novel) were identified. Among these, 23 variants were deemed likely pathogenic or pathogenic, while 16 were classified as variants of unknown significance (VUS). Out of the 38 different PIRD mutations, 22 were missense, eight were nonsense, five were in/dels, and one represented a single-base substitution in an exon–intron junction sequence, likely affecting a splice site.

This study marks the first instance of using next-generation sequencing within our country to investigate the distribution of mutations in the PIRD patient population. Turkey has reported a notably high rate of consanguineous marriages [20]. This phenomenon has contributed to a heightened prevalence of autosomal recessive inherited diseases, including Inborn Errors of Immunity. Among our cohort, recessive PIRD genes constituted 45% of cases, while dominant variants accounted for 40%, and X-linked PIRD genes constituted 15%.

In a Dutch cohort, NGS-based assessment for IEIs showed the highest yields among pediatric patients, within the immune dysregulation cluster, most patients received a diagnosis of familial hemophagocytic lymphohistiocytosis (HLH), with additional cases including autoimmune lymphoproliferative syndrome (ALPS), primarily attributed to pathogenic FAS variants [14]. Conversely, our cohort is primarily clustered within the immune dysregulation category, with a diagnosis of syndromes characterized by autoimmunity.

In a study from India, diseases involving immune dysregulation were observed in 20 patients. Most frequently, most of the patients are diagnosed with FHL [21].

In another study from Egypt, genetic assessments were conducted for 39 patients exhibiting immune dysregulation disorders. Among them, 21 individuals from 15 distinct consanguineous families displayed variations in the LRBA gene. Other infrequent genetic diagnoses included variants in IL10RA, IL10RB, FOXP3, AIRE, DOCK8, SLC7A7, UNC13D, PRKCD, SH2D1A, RIPK1, and FAS variants [22].

Our immune gene panel differs from the Dutch, Iranian, and Egyptian cohorts, as it involves a distinct panel lacking certain HLH genes found in the Primary Immune Deficiency Research Panel v2. Our clinicians preferred the HLH panel over the immune panel for FHL, which resulted in relatively lower FHL findings when assessed with our immune panel.

LRBA deficiency is an autosomal recessive disorder arising from biallelic mutations in the LRBA gene (OMIM #614,700). Clinically, it is characterized by early-onset hypogammaglobulinemia, autoimmune manifestations, susceptibility to inflammatory bowel disease, and recurrent infections. While partial isodisomy-associated LRBA deficiency has been reported previously [23], our study reports the first instance of LRBA mutation becoming homozygous through whole chromosome uniparental disomy (UPD).

Homozygous AIRE mutations c.769C > T (Arg257Ter), c.415C > T (p.Arg139Ter), and c.254A > G (p.Tyr85Cys) exhibit a founder effect in the Finnish, Sardinian, and Iranian Jewish populations, respectively [24]. The c.769C > T (Arg257Ter) variant in exon 6 has been identified in 89% of Finnish APECED alleles but is also the most prevalent across other ethnic groups. A literature review involving 23 published Turkish APECED patients revealed that the Finnish major mutation, c.769C > T (Arg257Ter), is prevalent in the Turkish population [24]. Of significance, three out of four (75%) APECED cases in our study featured the c.769C > T (Arg257Ter) variation.

Currently, there are nine genes associated with IEIs in which mutations have been detected for both loss-of-function and gain-of-function mutations: CFB, C3, CARD11, STAT1, STAT3, WAS, JAK1, IFIH1, and ZAP70 [2]. Gain-of-function (GOF) mutations bestow a different function upon the mutant gene due to the mutation it undergoes, leading to unexpected protein production. This type of mutation increases the transcription of a gene, endowing it with heightened activity and mobility, often referred to as a hypermorphic gene. Conversely, loss-of-function (LOF) mutations render the gene product dysfunctional. A gene product completely devoid of function is termed a null allele or amorphous allele. If the mutant type retains partial function, it is referred to as a hypomorphic allele. For instance, while functional mutations in STAT3 may manifest with lymphoproliferation, including lymphadenopathy and hepatosplenomegaly, and early-onset multisystem autoimmunity, STAT3 loss-of-function mutations underlie hyperimmunoglobulin E syndrome (Job’s syndrome). This syndrome is characterized by recurrent infections, eczema-like skin rashes, and vulnerability to severe lung infections. While both LOF and GOF of STAT3 cause immune deficiency, GOF leads to infections distinct from those observed with LOF, accompanied by more common connective tissue abnormalities [25, 26]. Moreover, a 5-year-old male with a novel ZAP70 c.1448C > T (p.Ser483Phe) variant underwent hematopoietic stem cell transplantation (HSCT), yielding successful clinical and immunologic outcomes.

CTLA4 deficiency is a rare disorder profoundly disrupting immune system regulation, leading to conditions such as intestinal disease, respiratory infections, autoimmune issues, and enlarged lymph nodes, liver, and spleen. Abatacept offers a potentially effective treatment for patients with documented CTLA-4 deficiency, inducing and sustaining remission of enteropathy [27].

In summary, PIRDs can manifest similar clinical profiles despite distinct genetic defects, and conversely, the same genetic defect can result in diverse clinical presentations. Beyond clinical diagnosis, identifying the molecular defect causing the disease is pivotal for prognosis prediction, treatment planning (e.g., abatacept, HSCT), preimplantation genetics, prenatal diagnosis, and carrier identification. Despite the genetic diversity underpinning PIRDs, genetic counseling assumes a crucial role in managing and shaping future decisions for affected families.

In conclusion, the integration of NGS into the study of PIRD molecular genetics provides a comprehensive and nuanced understanding of the disorder. From identifying rare variants to uncovering novel mutations, NGS emerges as a cornerstone technology, offering unprecedented insights that pave the way for personalized medicine and improved patient outcomes in the realm of Inborn Errors of Immunity. A key conclusion from this multicenter study: This report represents the inaugural utilization of NGS for diagnosing the Turkish PIRD cohort, offering novel insights that expand the spectrum of clinical manifestations attributed to various PIRD-related mutations.

## Data Availability

There is no applicable data available for this study.
